# Long-term weight loss effects of semaglutide in obesity without diabetes in the SELECT trial

**DOI:** 10.1038/s41591-024-02996-7

**Published:** 2024-05-13

**Authors:** Donna H. Ryan, Ildiko Lingvay, John Deanfield, Steven E. Kahn, Eric Barros, Bartolome Burguera, Helen M. Colhoun, Cintia Cercato, Dror Dicker, Deborah B. Horn, G. Kees Hovingh, Ole Kleist Jeppesen, Alexander Kokkinos, A. Michael Lincoff, Sebastian M. Meyhöfer, Tugce Kalayci Oral, Jorge Plutzky, André P. van Beek, John P. H. Wilding, Robert F. Kushner

**Affiliations:** 1 Pennington Biomedical Research Center, Baton Rouge, LA USA; 2 Department of Internal Medicine/Endocrinology and Peter O’ Donnell Jr. School of Public Health, University of Texas Southwestern Medical Center, Dallas, TX USA; 3 Institute of Cardiovascular Science, University College London, London, UK; 4 VA Puget Sound Health Care System and University of Washington, Seattle, WA USA; 5 Novo Nordisk A/S, Søborg, Denmark; 6 Endocrinology and Metabolism Institute, Cleveland Clinic, Cleveland, OH USA; 7 Institute of Genetics and Cancer, University of Edinburgh, Edinburgh, UK; 8 Obesity Unit, Department of Endocrinology, Hospital das Clínicas, University of São Paulo, São Paulo, Brazil; 9 Internal Medicine Department D, Hasharon Hospital-Rabin Medical Center, Faculty of Medicine, Tel Aviv University, Tel Aviv, Israel; 10 Center for Obesity Medicine and Metabolic Performance, Department of Surgery, University of Texas McGovern Medical School, Houston, TX USA; 11 First Department of Propaedeutic Internal Medicine, School of Medicine, National and Kapodistrian University of Athens, Athens, Greece; 12 Department of Cardiovascular Medicine, Cleveland Clinic, and Cleveland Clinic Lerner College of Medicine of Case Western Reserve University, Cleveland, OH USA; 13 Institute of Endocrinology & Diabetes, University of Lübeck, Lübeck, Germany; 14 Cardiovascular Division, Brigham and Women’s Hospital, Harvard Medical School, Boston, MA USA; 15 University of Groningen, University Medical Center Groningen, Department of Endocrinology, Groningen, the Netherlands; 16 Department of Cardiovascular and Metabolic Medicine, University of Liverpool, Liverpool, UK; 17 Northwestern University Feinberg School of Medicine, Chicago, IL USA

**Keywords:** Health care, Medical research

## Abstract

In the SELECT cardiovascular outcomes trial, semaglutide showed a 20% reduction in major adverse cardiovascular events in 17,604 adults with preexisting cardiovascular disease, overweight or obesity, without diabetes. Here in this prespecified analysis, we examined effects of semaglutide on weight and anthropometric outcomes, safety and tolerability by baseline body mass index (BMI). In patients treated with semaglutide, weight loss continued over 65 weeks and was sustained for up to 4 years. At 208 weeks, semaglutide was associated with mean reduction in weight (−10.2%), waist circumference (−7.7 cm) and waist-to-height ratio (−6.9%) versus placebo (−1.5%, −1.3 cm and −1.0%, respectively; *P* < 0.0001 for all comparisons versus placebo). Clinically meaningful weight loss occurred in both sexes and all races, body sizes and regions. Semaglutide was associated with fewer serious adverse events. For each BMI category (<30, 30 to <35, 35 to <40 and ≥40 kg m^−^^2^) there were lower rates (events per 100 years of observation) of serious adverse events with semaglutide (43.23, 43.54, 51.07 and 47.06 for semaglutide and 50.48, 49.66, 52.73 and 60.85 for placebo). Semaglutide was associated with increased rates of trial product discontinuation. Discontinuations increased as BMI class decreased. In SELECT, at 208 weeks, semaglutide produced clinically significant weight loss and improvements in anthropometric measurements versus placebo. Weight loss was sustained over 4 years. ClinicalTrials.gov identifier: NCT03574597.

## Abstract (web summary)

A prespecified analysis of the SELECT trial revealed that patients assigned to once-weekly subcutaneous semaglutide 2.4 mg lost significantly more weight than those receiving placebo and showed improvements in various anthropometric indices.

## Main

The worldwide obesity prevalence, defined by body mass index (BMI) ≥30 kg m^−^^2^, has nearly tripled since 1975 (ref. ^1^). BMI is a good surveillance measure for population changes over time, given its strong correlation with body fat amount on a population level, but it may not accurately indicate the amount or location of body fat at the individual level^2^. In fact, the World Health Organization defines clinical obesity as ‘abnormal or excessive fat accumulation that may impair health’^1^. Excess abnormal body fat, especially visceral adiposity and ectopic fat, is a driver of cardiovascular (CV) disease (CVD)^3–5^, and contributes to the global chronic disease burden of diabetes, chronic kidney disease, cancer and other chronic conditions^6,7^.

Remediating the adverse health effects of excess abnormal body fat through weight loss is a priority in addressing the global chronic disease burden. Improvements in CV risk factors, glycemia and quality-of-life measures including personal well-being and physical functioning generally begin with modest weight loss of 5%, whereas greater weight loss is associated with more improvement in these measures^8–10^. Producing and sustaining durable and clinically significant weight loss with lifestyle intervention alone has been challenging^11^. However, weight-management medications that modify appetite can make attaining and sustaining clinically meaningful weight loss of ≥10% more likely^12^. Recently, weight-management medications, particularly those comprising glucagon-like peptide-1 receptor agonists, that help people achieve greater and more sustainable weight loss have been developed^13^. Once-weekly subcutaneous semaglutide 2.4 mg, a glucagon-like peptide-1 receptor agonist, is approved for chronic weight management^14–16^ and at doses of up to 2.0 mg is approved for type 2 diabetes treatment^17–19^. In patients with type 2 diabetes and high CV risk, semaglutide at doses of 0.5 mg and 1.0 mg has been shown to significantly lower the risk of CV events^20^. The SELECT trial (Semaglutide Effects on Heart Disease and Stroke in Patients with Overweight or Obesity) studied patients with established CVD and overweight or obesity but without diabetes. In SELECT, semaglutide was associated with a 20% reduction in major adverse CV events (hazard ratio 0.80, 95% confidence interval (CI) 0.72 to 0.90; *P* < 0.001)^21^. Data derived from the SELECT trial offer the opportunity to evaluate the weight loss efficacy, in a geographically and racially diverse population, of semaglutide compared with placebo over 208 weeks when both are given in addition to standard-of-care recommendations for secondary CVD prevention (but without a focus on targeting weight loss). Furthermore, the data allow examination of changes in anthropometric measures such as BMI, waist circumference (WC) and waist-to-height ratio (WHtR) as surrogates for body fat amount and location^22,23^. The diverse population can also be evaluated for changes in sex- and race-specific ‘cutoff points’ for BMI and WC, which have been identified as anthropometric measures that predict cardiometabolic risk^8,22,23^.

This prespecified analysis of the SELECT trial investigated weight loss and changes in anthropometric indices in patients with established CVD and overweight or obesity without diabetes, who met inclusion and exclusion criteria, within a range of baseline categories for glycemia, renal function and body anthropometric measures.

## Results

### Study population

The SELECT study enrolled 17,604 patients (72.3% male) from 41 countries between October 2018 and March 2021, with a mean (s.d.) age of 61.6 (8.9) years and BMI of 33.3 (5.0) kg m^−^^2^ (ref. ^21^). The baseline characteristics of the population have been reported^24^. Supplementary Table 1 outlines SELECT patients according to baseline BMI categories. Of note, in the lower BMI categories (<30 kg m^−^^2^ (overweight) and 30 to <35 kg m^−^^2^ (class I obesity)), the proportion of Asian individuals was higher (14.5% and 7.4%, respectively) compared with the proportion of Asian individuals in the higher BMI categories (BMI 35 to <40 kg m^−^^2^ (class II obesity; 3.8%) and ≥40 kg m^−^^2^ (class III obesity; 2.2%), respectively). As the BMI categories increased, the proportion of women was higher: in the class III BMI category, 45.5% were female, compared with 20.8%, 25.7% and 33.0% in the overweight, class I and class II categories, respectively. Lower BMI categories were associated with a higher proportion of patients with normoglycemia and glycated hemoglobin <5.7%. Although the proportions of patients with high cholesterol and history of smoking were similar across BMI categories, the proportion of patients with high-sensitivity C-reactive protein ≥2.0 mg dl^−1^ increased as the BMI category increased. A high-sensitivity C-reactive protein >2.0 mg dl^−1^ was present in 36.4% of patients in the overweight BMI category, with a progressive increase to 43.3%, 57.3% and 72.0% for patients in the class I, II and III obesity categories, respectively.

### Weight and anthropometric outcomes

#### Percentage weight loss

**Fig. 1:**
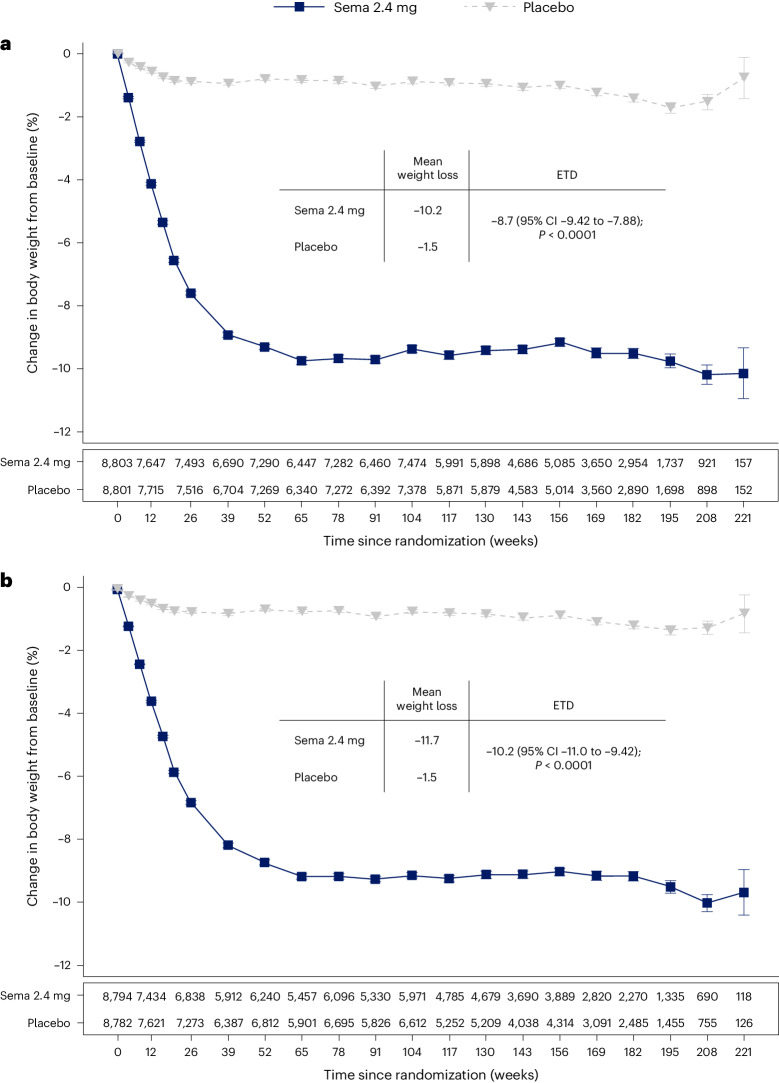
Percentage change in mean body weight from baseline through week 208 for all patients in-trial^21^ and first on-treatment. **a**,**b**, Observed data from the in-trial period (**a**) and first on-treatment (**b**). The symbols are the observed means, and error bars are ±s.e.m. Numbers shown below each panel represent the number of patients contributing to the means. Analysis of covariance with treatment and baseline values was used to estimate the treatment difference. Exact *P* values are 1.323762 × 10^−94^ and 9.80035 × 10^−100^ for **a** and **b**, respectively. *P* values are two-sided and are not adjusted for multiplicity. ETD, estimated treatment difference; sema, semaglutide.

The average percentage weight-loss trajectories with semaglutide and placebo over 4 years of observation are shown in Fig. 1a (ref. ^21^). For those in the semaglutide group, the weight-loss trajectory continued to week 65 and then was sustained for the study period through week 208 (−10.2% for the semaglutide group, −1.5% for the placebo group; treatment difference −8.7%; 95% CI −9.42 to −7.88; *P* < 0.0001). To estimate the treatment effect while on medication, we performed a first on-treatment analysis (observation period until the first time being off treatment for >35 days). At week 208, mean weight loss in the semaglutide group analyzed as first on-treatment was −11.7% compared with −1.5% for the placebo group (Fig. 1b; treatment difference −10.2%; 95% CI −11.0 to −9.42; *P* < 0.0001).

#### Categorical weight loss and individual body weight change

**Fig. 2:**
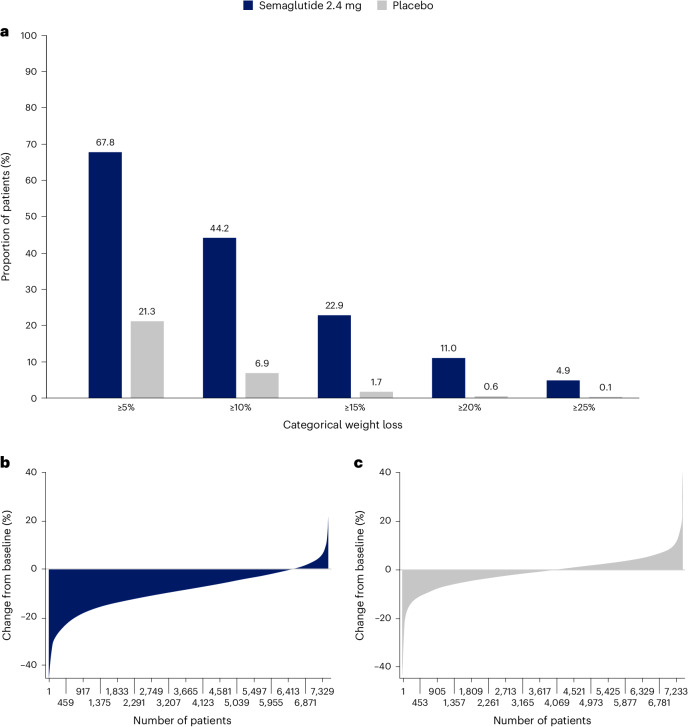
Variation in weight loss response for semaglutide andplacebo. **a**, Categorical weight loss from baseline at week 104 for semaglutide and placebo. Data from the in-trial period. Bars depict the proportion (%) of patients receiving semaglutide or placebo who achieved ≥5%, ≥10%, ≥15%, ≥20% and ≥25% weight loss. **b**,**c**, Percentage change in body weight for individual patients from baseline to week 104 for semaglutide (**b**) and placebo (**c**). Each patient’s percentage change in body weight is plotted as a single bar.

Among in-trial (intention-to-treat principle) patients at week 104, weight loss of ≥5%, ≥10%, ≥15%, ≥20% and ≥25% was achieved by 67.8%, 44.2%, 22.9%, 11.0% and 4.9%, respectively, of those treated with semaglutide compared with 21.3%, 6.9%, 1.7%, 0.6% and 0.1% of those receiving placebo (Fig. 2a). Individual weight changes at 104 weeks for the in-trial populations for semaglutide and placebo are depicted in Fig. 2b and Fig. 2c, respectively. These waterfall plots show the variation in weight-loss response that occurs with semaglutide and placebo and show that weight loss is more prominent with semaglutide than placebo.

#### Change in WC

WC change from baseline to 104 weeks has been reported previously in the primary outcome paper^21^. The trajectory of WC change mirrored that of the change in body weight. At week 208, average reduction in WC was −7.7 cm with semaglutide versus −1.3 cm with placebo, with a treatment difference of −6.4 cm (95% CI −7.18 to −5.61; *P* < 0.0001)^21^.

#### WC cutoff points

**Fig. 3:**
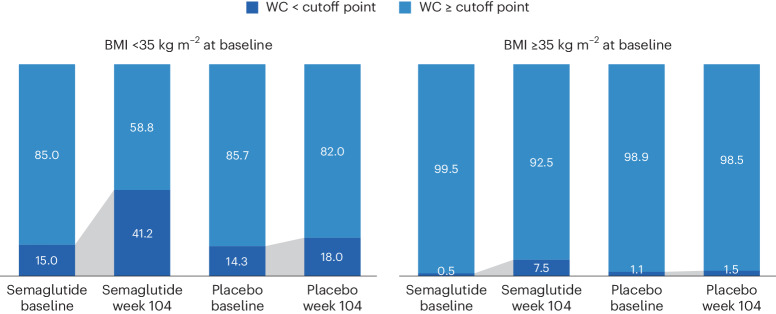
Proportion (%) of in-trial patients achieving sex- and race-specific WC cutoff points for increased metabolic risk according to baseline BMI <35 or ≥35 kg m^−^^2^. WC cutoff points; Asian women <80 cm, non-Asian women <88 cm, Asian men <88 cm, non-Asian men <102 cm.

We analyzed achievement of sex- and race-specific cutoff points for WC by BMI <35 kg m^−^^2^ or ≥35 kg m^−^^2^, because for BMI >35 kg m^−^^2^, WC is more difficult technically and, thus, less accurate as a risk predictor^4,25,26^. Within the SELECT population with BMI <35 kg m^−^^2^ at baseline, 15.0% and 14.3% of the semaglutide and placebo groups, respectively, were below the sex- and race-specific WC cutoff points. At week 104, 41.2% fell below the sex- and race-specific cutoff points for the semaglutide group, compared with only 18.0% for the placebo group (Fig. 3).

#### Waist-to-height ratio

At baseline, mean WHtR was 0.66 for the study population. The lowest tertile of the SELECT population at baseline had a mean WHtR <0.62, which is higher than the cutoff point of 0.5 used to indicate increased cardiometabolic risk^27^, suggesting that the trial population had high WCs. At week 208, in the group randomized to semaglutide, there was a relative reduction of 6.9% in WHtR compared with 1.0% in placebo (treatment difference −5.87% points; 95% CI −6.56 to −5.17; *P* < 0.0001).

#### BMI category change

**Fig. 4:**
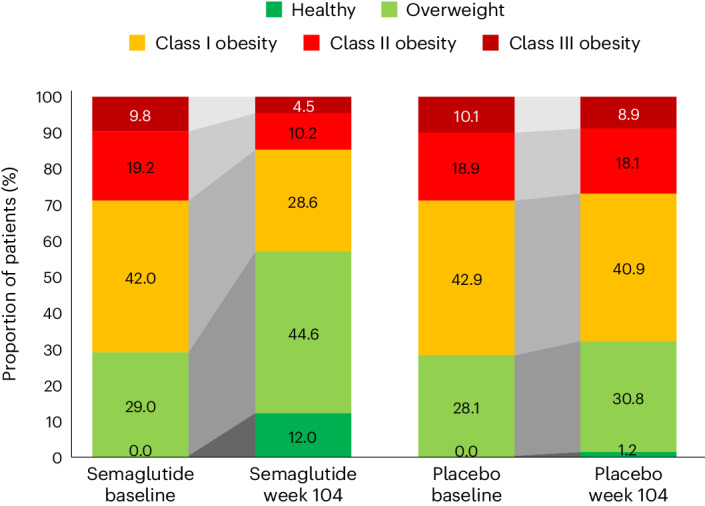
Change in BMI category (healthy, overweight, class I obesity, class II obesity and class III obesity) at baseline and week 104 for in-trial patients. In the semaglutide group, 12.0% of patients achieved normal weight status at week 104 (from 0% at baseline), compared with 1.2% (from 0% at baseline) for placebo. BMI classes: healthy (BMI <25 kg m^−^^2^), overweight (25 to <30 kg m^−^^2^), class I obesity (30 to <35 kg m^−^^2^), class II obesity (35 to <40 kg m^−^^2^) and class III obesity (BMI ≥40 kg m^−^^2^).

At week 104, 52.4% of patients treated with semaglutide achieved improvement in BMI category compared with 15.7% of those receiving placebo. Proportions of patients in the BMI categories at baseline and week 104 are shown in Fig. 4, which depicts in-trial patients receiving semaglutide and placebo. The BMI category change reflects the superior weight loss with semaglutide, which resulted in fewer patients being in the higher BMI categories after 104 weeks. In the semaglutide group, 12.0% of patients achieved a BMI <25 kg m^−^^2^, which is considered the healthy BMI category, compared with 1.2% for placebo; per study inclusion criteria, no patients were in this category at baseline. The proportion of patients with obesity (BMI ≥30 kg m^−^^2^) fell from 71.0% to 43.3% in the semaglutide group versus 71.9% to 67.9% in the placebo group.

### Weight and anthropometric outcomes by subgroups

**Fig. 5:**
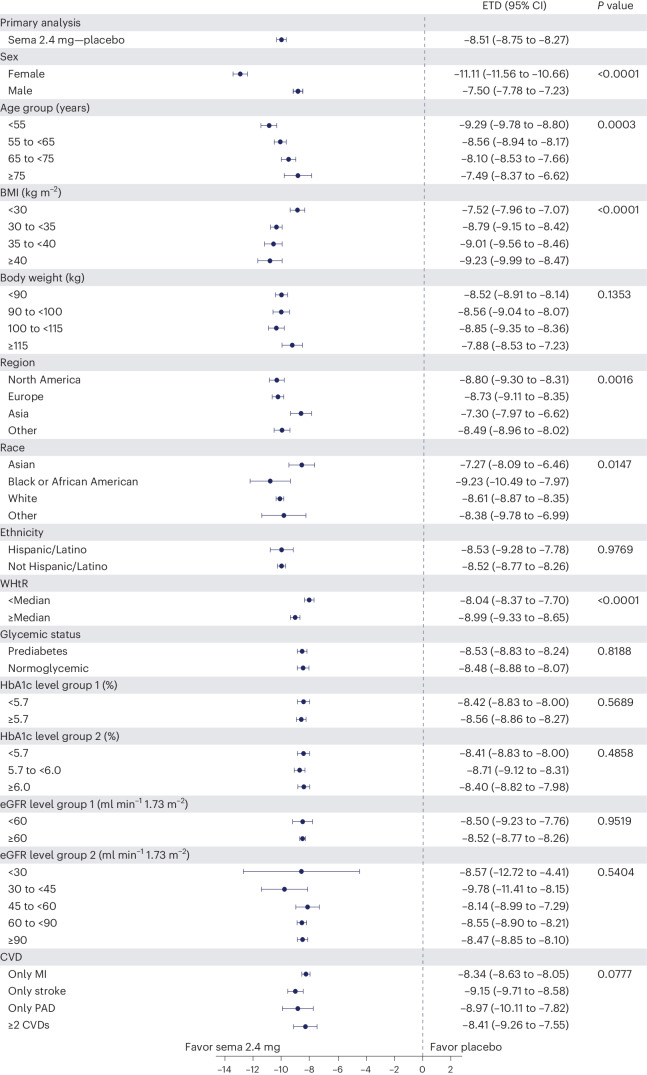
Effect of semaglutide treatment or placebo on mean percentage change in body weight from baseline to week 104 by subgroups. Data from the in-trial period. *N* = 17,604. *P* values represent test of no interaction effect. *P* values are two-sided and are not adjusted for multiplicity. The dots show estimated treatment differences, and the error bars show 95% CIs. Details of the statistical models are available in Methods. ETD, estimated treatment difference; HbA1c, glycated hemoglobin; MI, myocardial infarction; PAD, peripheral artery disease; sema, semaglutide.

**Extended Data Fig. 1:**
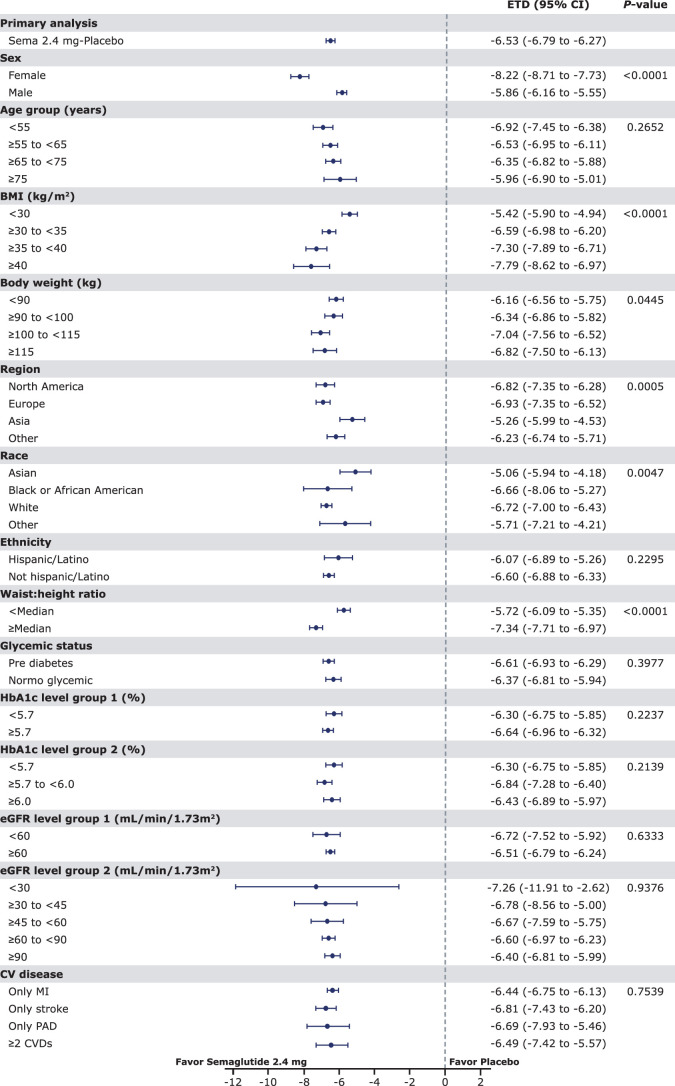
Effect of semaglutide treatment or placebo on waist circumference from baseline to week 104 by subgroups. Data from the in-trial period. *N* = 17,604. *P* values represent test of no interaction effect. *P* values are two-sided and not adjusted for multiplicity. The dots show estimated treatment differences and the error bars show 95% confidence intervals. Details of the statistical models are available in Methods. BMI, body mass index; CI, confidence interval; CV, cardiovascular; CVD, cardiovascular disease; eGFR, estimated glomerular filtration rate; ETD, estimated treatment difference; HbA1c, glycated hemoglobin; MI, myocardial infarction; PAD, peripheral artery disease; sema, semaglutide.

The forest plot illustrated in Fig. 5 displays mean body weight percentage change from baseline to week 104 for semaglutide relative to placebo in prespecified subgroups. Similar relationships are depicted for WC changes in prespecified subgroups shown in Extended Data Fig. 1. The effect of semaglutide (versus placebo) on mean percentage body weight loss as well as reduction in WC was found to be heterogeneous across several population subgroups. Women had a greater difference in mean weight loss with semaglutide versus placebo (−11.1% (95% CI −11.56 to −10.66) versus −7.5% in men (95% CI −7.78 to −7.23); *P* < 0.0001). There was a linear relationship between age category and degree of mean weight loss, with younger age being associated with progressively greater mean weight loss, but the actual mean difference by age group is small. Similarly, BMI category had small, although statistically significant, associations. Those with WHtR less than the median experienced slightly lower mean body weight change than those above the median, with estimated treatment differences −8.04% (95% CI −8.37 to −7.70) and −8.99% (95% CI −9.33 to −8.65), respectively (*P* < 0.0001). Patients from Asia and of Asian race experienced slightly lower mean weight loss (estimated treatment difference with semaglutide for Asian race −7.27% (95% CI −8.09 to −6.46; *P* = 0.0147) and for Asia −7.30 (95% CI −7.97 to −6.62; *P* = 0.0016)). There was no difference in weight loss with semaglutide associated with ethnicity (estimated treatment difference for Hispanic −8.53% (95% CI −9.28 to −7.76) or non-Hispanic −8.52% (95% CI −8.77 to 8.26); *P* = 0.9769), glycemic status (estimated treatment difference for prediabetes −8.53% (95% CI −8.83 to −8.24) or normoglycemia −8.48% (95% CI −8.88 to −8.07; *P* = 0.8188) or renal function (estimated treatment difference for estimated glomerular filtration rate (eGFR) <60 or ≥60 ml min^−1^ 1.73 m^−^^2^ being −8.50% (95% CI −9.23 to −7.76) and −8.52% (95% CI −8.77 to −8.26), respectively (*P* = 0.9519)).

### Safety and tolerability according to baseline BMI category

**Fig. 6:**
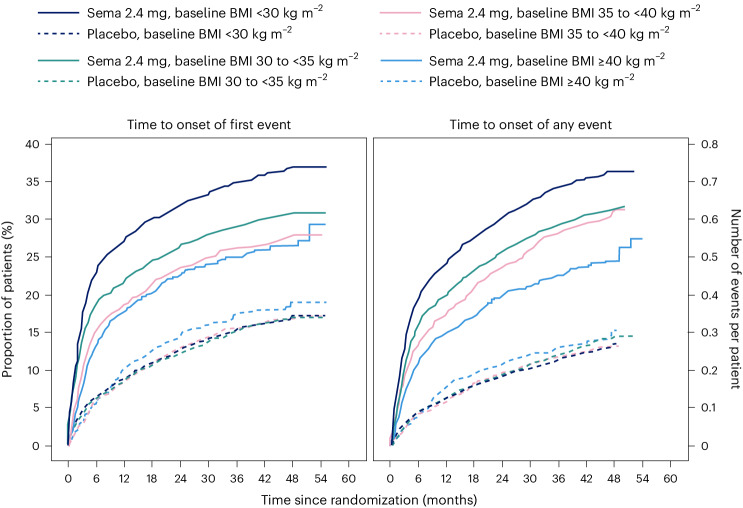
AEs leading to trial product discontinuation for baseline BMI class (<30 kg m^−^^2^, 30 to <35 kg m^−^^2^, 35 to <40 kg m^−^^2^, and ≥40 kg m^−^^2^); cumulative incidence for first event over time (left) and cumulative mean number of events over time (right). Data are in-trial from the full analysis set. sema, semaglutide.

We reported in the primary outcome of the SELECT trial that adverse events (AEs) leading to permanent discontinuation of the trial product occurred in 1,461 patients (16.6%) in the semaglutide group and 718 patients (8.2%) in the placebo group (*P* < 0.001)^21^. For this analysis, we evaluated the cumulative incidence of AEs leading to trial product discontinuation by treatment assignment and by BMI category (Fig. 6). For this analysis, with death modeled as a competing risk, we tracked the proportion of in-trial patients for whom drug was withdrawn or interrupted for the first time (Fig. 6, left) or cumulative discontinuations (Fig. 6, right). Both panels of Fig. 6 depict a graded increase in the proportion discontinuing semaglutide, but not placebo. For lower BMI classes, discontinuation rates are higher in the semaglutide group but not the placebo group.

We reported in the primary SELECT analysis that serious adverse events (SAEs) were reported by 2,941 patients (33.4%) in the semaglutide arm and by 3,204 patients (36.4%) in the placebo arm (*P* < 0.001)^21^. For this study, we analyzed SAE rates by person-years of treatment exposure for BMI classes (<30 kg m^−^^2^, 30 to <35 kg m^−^^2^, 35 to <40 kg m^−^^2^, and ≥40 kg m^−^^2^) and provide these data in Supplementary Table 2. We also provide an analysis of the most common categories of SAEs. Semaglutide was associated with lower SAEs, primarily driven by CV event and infections. Within each obesity class (<30 kg m^−^^2^, 30 to <35 kg m^−^^2^, 35 to <40 kg m^−^^2^, and ≥40 kg m^−^^2^), there were fewer SAEs in the group receiving semaglutide compared with placebo. Rates (events per 100 years of observation) of SAEs were 43.23, 43.54, 51.07 and 47.06 for semaglutide and 50.48, 49.66, 52.73 and 60.85 for placebo, with no evidence of heterogeneity. There was no detectable difference in hepatobiliary or gastrointestinal SAEs comparing semaglutide with placebo in any of the four BMI classes we evaluated.

## Discussion

The analyses of weight effects of the SELECT study presented here reveal that patients assigned to once-weekly subcutaneous semaglutide 2.4 mg lost significantly more weight than those receiving placebo. The weight-loss trajectory with semaglutide occurred over 65 weeks and was sustained up to 4 years. Likewise, there were similar improvements in the semaglutide group for anthropometrics (WC and WHtR). The weight loss was associated with a greater proportion of patients receiving semaglutide achieving improvement in BMI category, healthy BMI (<25 kg m^−^^2^) and falling below the WC cutoff point above which increased cardiometabolic risk for the sex and race is greater^22,23^. Furthermore, both sexes, all races, all body sizes and those from all geographic regions were able to achieve clinically meaningful weight loss. There was no evidence of increased SAEs based on BMI categories, although lower BMI category was associated with increased rates of trial product discontinuation, probably reflecting exposure to a higher level of drug in lower BMI categories. These data, representing the longest clinical trial of the effects of semaglutide versus placebo on weight, establish the safety and durability of semaglutide effects on weight loss and maintenance in a geographically and racially diverse population of adult men and women with overweight and obesity but not diabetes. The implications of weight loss of this degree in such a diverse population suggests that it may be possible to impact the public health burden of the multiple morbidities associated with obesity. Although our trial focused on CV events, many chronic diseases would benefit from effective weight management^28^.

There were variations in the weight-loss response. Individual changes in body weight with semaglutide and placebo were striking; still, 67.8% achieved 5% or more weight loss and 44.2% achieved 10% weight loss with semaglutide at 2 years, compared with 21.3% and 6.9%, respectively, for those receiving placebo. Our first on-treatment analysis demonstrated that those on-drug lost more weight than those in-trial, confirming the effect of drug exposure. With semaglutide, lower BMI was associated with less percentage weight loss, and women lost more weight on average than men (−11.1% versus −7.5% treatment difference from placebo); however, in all cases, clinically meaningful mean weight loss was achieved. Although Asian patients lost less weight on average than patients of other races (−7.3% more than placebo), Asian patients were more likely to be in the lowest BMI category (<30 kg m^−^^2^), which is known to be associated with less weight loss, as discussed below. Clinically meaningful weight loss was evident in the semaglutide group within a broad range of baseline categories for glycemia and body anthropometrics. Interestingly, at 2 years, a significant proportion of the semaglutide-treated group fell below the sex- and race-specific WC cutoff points, especially in those with BMI <35 kg m^−^^2^, and a notable proportion (12.0%) fell below the BMI cutoff point of 25 kg m^−^^2^, which is deemed a healthy BMI in those without unintentional weight loss. As more robust weight loss is possible with newer medications, achieving and maintaining these cutoff point targets may become important benchmarks for tracking responses.

The overall safety profile did not reveal any new signals from prior studies, and there were no BMI category-related associations with AE reporting. The analysis did reveal that tolerability may differ among specific BMI classes, since more discontinuations occurred with semaglutide among lower BMI classes. Potential contributors may include a possibility of higher drug exposure in lower BMI classes, although other explanations, including differences in motivation and cultural mores regarding body size, cannot be excluded.

Is the weight loss in SELECT less than expected based on prior studies with the drug? In STEP 1, a large phase 3 study of once-weekly subcutaneous semaglutide 2.4 mg in individuals without diabetes but with BMI >30 kg m^−^^2^ or 27 kg m^−^^2^ with at least one obesity-related comorbidity, the mean weight loss was −14.9% at week 68, compared with −2.4% with placebo^14^. Several reasons may explain the observation that the mean treatment difference was −12.5% in STEP 1 and −8.7% in SELECT. First, SELECT was designed as a CV outcomes trial and not a weight-loss trial, and weight loss was only a supportive secondary endpoint in the trial design. Patients in STEP 1 were desirous of weight loss as a reason for study participation and received structured lifestyle intervention (which included a −500 kcal per day diet with 150 min per week of physical activity). In the SELECT trial, patients did not enroll for the specific purpose of weight loss and received standard of care covering management of CV risk factors, including medical treatment and healthy lifestyle counseling, but without a specific focus on weight loss. Second, the respective study populations were quite different, with STEP 1 including a younger, healthier population with more women (73.1% of the semaglutide arm in STEP 1 versus 27.7% in SELECT) and higher mean BMI (37.8 kg m^−^^2^ versus 33.3 kg m^−^^2^, respectively)^14,21^. Third, major differences existed between the respective trial protocols. Patients in the semaglutide treatment arm of STEP 1 were more likely to be exposed to the medication at the full dose of 2.4 mg than those in SELECT. In SELECT, investigators were allowed to slow, decrease or pause treatment. By 104 weeks, approximately 77% of SELECT patients on dose were receiving the target semaglutide 2.4 mg weekly dose, which is lower than the corresponding proportion of patients in STEP 1 (89.6% were receiving the target dose at week 68)^14,21^. Indeed, in our first on-treatment analysis at week 208, weight loss was greater (−11.7% for semaglutide) compared with the in-trial analysis (−10.2% for semaglutide). Taken together, all these issues make less weight loss an expected finding in SELECT, compared with STEP 1.

The SELECT study has some limitations. First, SELECT was not a primary prevention trial, and the data should not be extrapolated to all individuals with overweight and obesity to prevent major adverse CV events. Although the data set is rich in numbers and diversity, it does not have the numbers of individuals in racial subgroups that may have revealed potential differential effects. SELECT also did not include individuals who have excess abnormal body fat but a BMI <27 kg m^−^^2^. Not all individuals with increased CV risk have BMI ≥27 kg m^−^^2^. Thus, the study did not include Asian patients who qualify for treatment with obesity medications at lower BMI and WC cutoff points according to guidelines in their countries^29^. We observed that Asian patients were less likely to be in the higher BMI categories of SELECT and that the population of those with BMI <30 kg m^−^^2^ had a higher percentage of Asian race. Asian individuals would probably benefit from weight loss and medication approaches undertaken at lower BMI levels in the secondary prevention of CVD. Future studies should evaluate CV risk reduction in Asian individuals with high CV risk and BMI <27 kg m^−^^2^. Another limitation is the lack of information on body composition, beyond the anthropometric measures we used. It would be meaningful to have quantitation of fat mass, lean mass and muscle mass, especially given the wide range of body size in the SELECT population.

An interesting observation from this SELECT weight loss data is that when BMI is ≤30 kg m^−^^2^, weight loss on a percentage basis is less than that observed across higher classes of BMI severity. Furthermore, as BMI exceeds 30 kg m^−^^2^, weight loss amounts are more similar for class I, II and III obesity. This was also observed in Look AHEAD, a lifestyle intervention study for weight loss^30^. The proportion (percentage) of weight loss seems to be less, on average, in the BMI <30 kg m^−^^2^ category relative to higher BMI categories, despite their receiving of the same treatment and even potentially higher exposure to the drug for weight loss^30^. Weight loss cannot continue indefinitely. There is a plateau of weight that occurs after weight loss with all treatments for weight management. This plateau has been termed the ‘set point’ or ‘settling point’, a body weight that is in harmony with the genetic and environmental determinants of body weight and adiposity^31^. Perhaps persons with BMI <30 kg m^−^^2^ are closer to their settling point and have less weight to lose to reach it. Furthermore, the cardiometabolic benefits of weight loss are driven by reduction in the abnormal ectopic and visceral depots of fat, not by reduction of subcutaneous fat stores in the hips and thighs. The phenotype of cardiometabolic disease but lower BMI (<30 kg m^−^^2^) may be one where reduction of excess abnormal and dysfunctional body fat does not require as much body mass reduction to achieve health improvement. We suspect this may be the case and suggest further studies to explore this aspect of weight-loss physiology.

In conclusion, this analysis of the SELECT study supports the broad use of once-weekly subcutaneous semaglutide 2.4 mg as an aid to CV event reduction in individuals with overweight or obesity without diabetes but with preexisting CVD. Semaglutide 2.4 mg safely and effectively produced clinically significant weight loss in all subgroups based on age, sex, race, glycemia, renal function and anthropometric categories. Furthermore, the weight loss was sustained over 4 years during the trial.

## Methods

### Trial design and participants

The current work complies with all relevant ethical regulations and reports a prespecified analysis of the randomized, double-blind, placebo-controlled SELECT trial (NCT03574597), details of which have been reported in papers describing study design and rationale^32^, baseline characteristics^24^ and the primary outcome^21^. SELECT evaluated once-weekly subcutaneous semaglutide 2.4 mg versus placebo to reduce the risk of major adverse cardiac events (a composite endpoint comprising CV death, nonfatal myocardial infarction or nonfatal stroke) in individuals with established CVD and overweight or obesity, without diabetes. The protocol for SELECT was approved by national and institutional regulatory and ethical authorities in each participating country. All patients provided written informed consent before beginning any trial-specific activity. Eligible patients were aged ≥45 years, with a BMI of ≥27 kg m^−^^2^ and established CVD defined as at least one of the following: prior myocardial infarction, prior ischemic or hemorrhagic stroke, or symptomatic peripheral artery disease. Additional inclusion and exclusion criteria can be found elsewhere^32^.

### Human participants research

The trial protocol was designed by the trial sponsor, Novo Nordisk, and the academic Steering Committee. A global expert panel of physician leaders in participating countries advised on regional operational issues. National and institutional regulatory and ethical authorities approved the protocol, and all patients provided written informed consent.

### Study intervention and patient management

Patients were randomly assigned in a double-blind manner and 1:1 ratio to receive once-weekly subcutaneous semaglutide 2.4 mg or placebo. The starting dose was 0.24 mg once weekly, with dose increases every 4 weeks (to doses of 0.5, 1.0, 1.7 and 2.4 mg per week) until the target dose of 2.4 mg was reached after 16 weeks. Patients who were unable to tolerate dose escalation due to AEs could be managed by extension of dose-escalation intervals, treatment pauses or maintenance at doses below the 2.4 mg per week target dose. Investigators were allowed to reduce the dose of study product if tolerability issues arose. Investigators were provided with guidelines for, and encouraged to follow, evidence-based recommendations for medical treatment and lifestyle counseling to optimize management of underlying CVD as part of the standard of care. The lifestyle counseling was not targeted at weight loss. Additional intervention descriptions are available^32^.

### Sex, race, body weight, height and WC measurements

Sex and race were self-reported. Body weight was measured without shoes and only wearing light clothing; it was measured on a digital scale and recorded in kilograms or pounds (one decimal with a precision of 0.1 kg or lb), with preference for using the same scale throughout the trial. The scale was calibrated yearly as a minimum unless the manufacturer certified that calibration of the weight scales was valid for the lifetime of the scale. Height was measured without shoes in centimeters or inches (one decimal with a precision of 0.1 cm or inches). At screening, BMI was calculated by the electronic case report form. WC was defined as the abdominal circumference located midway between the lower rib margin and the iliac crest. Measures were obtained in a standing position with a nonstretchable measuring tape and to the nearest centimeter or inch. The patient was asked to breathe normally. The tape touched the skin but did not compress soft tissue, and twists in the tape were avoided.

### Endpoints

The following endpoints relevant to this paper were assessed at randomization (week 0) to years 2, 3 and 4: change in body weight (%); proportion achieving weight loss ≥5%, ≥10%, ≥15% and ≥20%; change in WC (cm); and percentage change in WHtR (cm cm^−1^). Improvement in BMI category (defined as being in a lower BMI class) was assessed at week 104 compared with baseline according to BMI classes: healthy (BMI <25 kg m^−^^2^), overweight (25 to <30 kg m^−^^2^), class I obesity (30 to <35 kg m^−^^2^), class II obesity (35 to <40 kg m^−^^2^) and class III obesity (≥40 kg m^−^^2^). The proportions of individuals with BMI <35 or ≥35 kg m^−^^2^ who achieved sex- and race-specific cutoff points for WC (indicating increased metabolic risk) were evaluated at week 104. The WC cutoff points were as follows: Asian women <80 cm, non-Asian women <88 cm, Asian men <88 cm and non-Asian men <102 cm.

Overall, 97.1% of the semaglutide group and 96.8% of the placebo group completed the trial. During the study, 30.6% of those assigned to semaglutide did not complete drug treatment, compared with 27.0% for placebo.

### Statistical analysis

The statistical analyses for the in-trial period were based on the intention-to-treat principle and included all randomized patients irrespective of adherence to semaglutide or placebo or changes to background medications. Continuous endpoints were analyzed using an analysis of covariance model with treatment as a fixed factor and baseline value of the endpoint as a covariate. Missing data at the landmark visit, for example, week 104, were imputed using a multiple imputation model and done separately for each treatment arm and included baseline value as a covariate and fit to patients having an observed data point (irrespective of adherence to randomized treatment) at week 104. The fit model is used to impute values for all patients with missing data at week 104 to create 500 complete data sets. Rubin’s rules were used to combine the results. Estimated means are provided with s.e.m., and estimated treatment differences are provided with 95% CI. Binary endpoints were analyzed using logistic regression with treatment and baseline value as a covariate, where missing data were imputed by first using multiple imputation as described above and then categorizing the imputed data according to the endpoint, for example, body weight percentage change at week 104 of <0%. Subgroup analyses for continuous and binary endpoints also included the subgroup and interaction between treatment and subgroup as fixed factors. Because some patients in both arms continued to be followed but were off treatment, we also analyzed weight loss by first on-treatment group (observation period until first time being off treatment for >35 days) to assess a more realistic picture of weight loss in those adhering to treatment. CIs were not adjusted for multiplicity and should therefore not be used to infer definitive treatment effects. All statistical analyses were performed with SAS software, version 9.4 TS1M5 (SAS Institute).

### Reporting summary

Further information on research design is available in the Nature Portfolio Reporting Summary linked to this article.

## Online content

Any methods, additional references, Nature Portfolio reporting summaries, source data, extended data, supplementary information, acknowledgements, peer review information; details of author contributions and competing interests; and statements of data and code availability are available at 10.1038/s41591-024-02996-7.

### Supplementary information

Reporting Summary

Supplementary Tables 1 and 2 Supplementary Table 1. Baseline characteristics by BMI class. Data are represented as number and percentage of patients. Renal function categories were based on the eGFR as per Chronic Kidney Disease Epidemiology Collaboration. Albuminuria categories were based on UACR. Smoking was defined as smoking at least one cigarette or equivalent daily. The category ‘Other’ for CV inclusion criteria includes patients where it is unknown if the patient fulfilled only one or several criteria and patients who were randomized in error and did not fulfill any criteria. Supplementary Table 2. SAEs according to baseline BMI category. *P* value: two-sided *P* value from Fisher’s exact test for test of no difference.

## Data Availability

Data will be shared with bona fide researchers who submit a research proposal approved by the independent review board. Individual patient data will be shared in data sets in a deidentified and anonymized format. Information about data access request proposals can be found at https://www.novonordisk-trials.com/.
